# Authors’ Response to Peer Reviews of “Chaotic and Stochastic Components in an Influenza Surveillance Series: Nonlinear Dynamics and Predictive Modeling Study”

**DOI:** 10.2196/101688

**Published:** 2026-06-05

**Authors:** Carlos Pedro dos Santos Goncalves, Carlos Rouco

**Affiliations:** 1 ECEO - School of Organisational and Economic Sciences - Civil Aviation and Airport Management Department, Lusófona University, Intrepid Lab Hub of Lusófona University at CETRAD, Campo Grande, 376, Lisbon, 1749-024, Portugal, 351 217 515 500

**Keywords:** epidemiology, epidemiologic methods, epidemiological monitoring, chaos theory, topological data analysis, autoregressive conditional heteroskedasticity

*This is the authors’ response to peer-review reports for “Chaotic and Stochastic Components in an Influenza Surveillance Series: Nonlinear Dynamics and Predictive Modeling Study.”*

## Round 1 Review

### Reviewer F [1]

#### General Comments

*This paper [2] presents an interesting application of chaos theory, nonlinear time series analysis, and topological machine learning to influenza epidemiological data. The methodology is strong and promising. However, the manuscript requires substantial improvements to enhance clarity and accessibility.*

**Response:** Thank you for the valuable comments. The article has been extensively revised to address the main points raised by the reviewer.

The title was revised to better fit the article’s research objectives.

#### Specific Comments

##### Major Comments

*1. Abstract: Currently very dense, heavy with jargon, and difficult for nonspecialists to follow. Please simplify sentences and clearly highlight the main objective, methodology, key findings, and implications*

**Response:** The abstract was revised with a structured background referring to the context of the study, the objective, the methods used, the results achieved, and the conclusions that are addressed in detail in the article in connection with epidemiological risk analysis and surveillance.

*2. Introduction: Starts too technically. Begin with a motivation, for instance, “Why chaos theory in epidemiology?” and then gradually introduce deterministic vs stochastic chaos. Also, state the study’s aim more explicitly.*

**Response:** The Introduction has been fully revised to introduce the concept of chaos and its relevance in epidemiology, providing a historical review of theoretical and empirical studies into chaos in epidemiology, linking these studies to the major implications of chaotic dynamics for epidemiology and the context and motivation for the article.

*3. Methods: The section is lengthy, with equations embedded in the text. Consider dividing into subsections for readability. Moving some theoretical background to an appendix would also improve flow. Please add a figure summarizing the main steps of the study.*

**Response:** The article has two major objectives: the reinforcement of empirical research on possible chaotic dynamics in influenza and the study of possible complex dynamical noise affecting those chaotic dynamics, addressing the consequences for epidemiological risk analysis and surveillance.

To that end, an epidemiological surveillance series is used, and a recent topological method for decomposition between chaotic and stochastic dynamics is employed. We now provide the references for the method that was first developed and applied to air traffic data and the study of the impact of COVID-19 at an artificial intelligence conference presentation and then for sunspot data.

In the revised article, a modification to the method was introduced in the last stage to make it more compatible with epidemiological forecasting, surveillance, and risk analysis.

In this way, the Methods section now provides a general overview of the method, including the modification that was introduced in the revised article, with the description of the three stages on which the method is built and the aims and types of analyses done at each stage.

*4. Results: The analysis is rich but scattered. Figures and tables are described without sufficient interpretation. For each, explicitly state the following: What do we see? What does it mean? Why does it matter?*

**Response:** The Results section is extensively revised with a division into four subsections: the first subsection presents the epidemiological series and performs the power spectrum analysis of the time series, and the remaining three subsections correspond to the implementation of the method.

Given the refocusing of the method for a direct integration into epidemiological surveillance and risk analysis, several topological analyses were dropped, with just the essential analyses remaining for identifying and characterizing the profile of both the chaotic and stochastic dynamics and how they link back to epidemiological processes and to epidemiological surveillance.

The implications of the results for the theoretical and empirical research into chaos in epidemiology are addressed in both the Results section and in a wider context in the Discussion section in a way that links back to the Introduction.

The revised article now focuses specifically on the evidence of stochastic chaos in influenza in comparison with the theoretical and empirical literature on chaos in epidemiology and the implications for epidemiological surveillance and risk analysis.

Chaos in epidemiology has mostly been developed within the theoretical framework of epidemiological modeling, with models showing the possibility of chaos.

However, the major rise in empirical research on chaos in surveillance data was during the COVID-19 crisis, focusing on SARS-CoV-2. The main objective of this research was to find markers of chaos and characterize the attractors rather than a full analysis of the chaotic plus stochastic process.

The findings of this article are thus discussed in connection with both the SARS-CoV-2 pattern and influenza studies, thus providing a direct link to previous empirical epidemiological studies on chaos that have found evidence of chaos in epidemiological surveillance data.

The novelty of this article is the decomposition of the target surveillance series’ dynamics down to independent and identically distributed (IID) noise and the identification of the main dynamical patterns connecting them to epidemiological processes while also providing for a machine learning scheme that can be used for epidemiological surveillance. These two dimensions of this article are addressed in both the Results and Discussion sections.

Specifically, since we found a chaotic process affecting the mean and a nonstationary variance that can be fully captured by an autoregressive conditional heteroskedasticity (ARCH) model, we obtained direct implications for epidemiological risk analysis on both the prediction and the use of risk metrics such as variance and outbreak probability that are not stationary. The link between these two processes and epidemiology is being researched.

The implications for theoretical modeling in epidemiology are also addressed in the sense that these models may need to incorporate a stochastic component that is not Gaussian white noise but rather an ARCH process to account for the two components: the long wave intermittent chaotic and the turbulent ARCH, leading to a stochastic chaotic mean and a nonstationary variance.

We also included an additional analysis that involves multiperiod predictability (multiweek) and discusses its implications for epidemiological surveillance.

Some relevant equations are introduced in the Results section, which are necessary to understand the stochastic ARCH modeling used, but the more technical components regarding the topological embedding for attractor reconstruction and the different ARCH models tested are supplied in Appendices A and B, respectively.

Since the key topological insights can be drawn from the *k*-nearest neighbors machine learning algorithm applied for the attractor reconstruction, we describe in detail in Appendix A how the *k*-nearest neighbors algorithm works and removed other nonessential topological data analysis methods in the text to make it more focused on the main objectives of the work.

*5. Discussion: A separate Discussion section would strengthen the paper. You can structure it as follows: evidence of stochastic chaos in influenza, implications for epidemiological modeling and prediction, and comparison with previous applications of chaos theory.*

**Response:** The Discussion section addresses the wider context for the article and the implications of the work for that wider context now featuring the following subsections for better readability and understanding of these implications:

- Implications for Theoretical Studies Into Chaos in Epidemiology
- Empirical Implications
- Implications for Epidemiological Risk Analysis and Surveillance
- Limitations and Future Work

### Reviewer FU [3]

*The manuscript under review presents an empirical methodology for studying stochastic chaos in epidemiological data by combining topological data analysis, topological machine learning, and nonlinear time series analysis to decompose influenza dynamics into deterministic chaotic and stochastic components down to the noise of IID random variables.*

**Response:** Thank you for the valuable comments. The article has been revised on key points, considering the three points raised by the reviewer as explained below.

*I recommend that the authors address the following shortcomings to improve the manuscript before publication.*

*1. The estimated largest Lyapunov exponent is approximately 0.001, which the authors characterize as “weak chaos.” However, such low values may be statistically indistinguishable from colored noise or stochastic processes with long-range dependencies. The authors should conduct additional tests using surrogate data to reliably confirm the deterministic chaotic nature of the reconstructed attractor.*

**Response:** Surrogate tests are now applied to the original attractor and the noise-filtered attractor using both the *k*-nearest neighbors predictions as a noise filter, as explained above, and using a wavelet filter for comparison. Surrogate data are produced with Monte Carlo simulation, calculating bootstrap *P* values for both a full power law stochastic process with the same decay and for a stochastic iterative amplitude-adjusted Fourier transform–generated signal, which is more adapted than the original iterative amplitude-adjusted Fourier transform when dealing with long memory stochastic processes with different spectral features and better captures the spectrum of this series. In all of the tests, the largest Lyapunov exponents are found to be strongly statistically significant, with the noise-filtered attractors being statistically significant at the 1% level.

*2. The authors should elaborate on how identifying stochastic chaos improves decision-relevant forecasting compared to established time series models in disease outbreak scenarios, including a discussion of lead times, calibration, and uncertainty quantification.*

**Response:** Established time series forecasting models are now used in the decomposition stage, the last stage of the method, involving different families of forecasters that not only provide insights into the epidemiological dynamics but also allow us to find how different types of forecasters work for different horizons. The following models are now tested on forecasting up to 5 steps ahead (5 weeks ahead).

- *k*-nearest neighbors: for analysis of the degree to which the topological structure of the reconstructed attractor contains exploitable topological information for forecasting
- Support vector machines: to evaluate the kernel family of forecasters with the radial basis function, linear, sigmoid, and polynomial of degree two and three tested
- Ridge regression: to evaluate linear approximations with adaptive autoregressive component
- Ensemble-based models: random forests and gradient boosting regressors

The linear ridge regression with penalty 0, when using the phase space reconstruction, is directly equivalent to a rolling window autoregressive process due to the fact that the delay embedding involves past values of the main series. The only issue is that since the attractor is strongly nonlinear and turbulent in the mean process and has nonstationary variance and heteroskedasticity with heavy tails even when removing the ARCH component, the autoregressive parameters are dynamical rather than fixed, and Gaussian likelihood methods are statistically incorrect for the current case.

On one step ahead (1 week) and two steps ahead (2 weeks) forecasting, the adaptive linear forecaster using linear ridge regression with penalty 0.5 is the best performer, with *R*^2^ scores of 92.11% (1 week ahead) and 85.95% (2 weeks ahead) forecasting. After that point, the gradient boosting regressor model performs better.

The rolling window ridge regressor is only a local adaptive linear approximation of the nonlinear attractor, which makes its coefficients actually dynamical. This reinforces the nonlinearity of the dynamics, as explained in the article. In this case, the major dynamical coefficients, rather than being stable, fluctuate significantly, which shows the nonlinearity, but they do so in an epidemiologically meaningful way that leads to insights into the overall dynamics.

Only a rolling window is applied since a full sample estimation would defeat the purpose of having a locally adaptive test of the ability of the attractor to support adaptive forecasting for epidemiological surveillance, which is the modification done.

However, the exponentially weighted moving average (EWMA) method that is applied to capture the dynamical variance in the stochastic process is tested against the major full ARCH time series models, which allows us to test the main time series models used when dealing with nonstationary variance. It turns out that none of these models is able to capture the full process down to IID, while the rolling window ridge regressor plus EWMA model is.

On another note, since all models are using a rolling window framework with out-of-sample prediction, that is, they are trained in a rolling window and used to predict new data adapting to the attractor’s epochs and to structural shocks like the impact of COVID-19’s period on influenza, all of the reported *R*^2^ scores are now forecasting scores on out-of-sample data, which did not take place in the original article in which the final *R*^2^ score was a goodness-of-fit score.

The link between major epidemiological risk metrics and underlying epidemiological dynamics is addressed in greater detail in the article, namely, the SD, which in this case depends specifically on the ARCH dynamics, and the outbreak probability, which depends on both the ARCH dynamics and the chaotic process in the mean. The implications of the results are addressed in the Discussion section in relation to both the epidemiological literature on chaos in epidemiological modeling and in relation to other empirical works developed around COVID-19 and influenza. This literature is now also reviewed in detail in the Introduction section for further context.

Two appendices were added to the work, including one explaining the phase space predictive topological reconstruction using *k*-nearest neighbors, which comes from the original method that was initially applied to air traffic and sunspot data, and that is now reviewed in the Introduction section for more detailed context.

The role of the *k*-nearest neighbors algorithm, as is now explained in the first appendix, is linked to the topological structure of nonlinear dynamics and chaos, where recurrence patterns leave a predictive structure that can be captured upon appropriate embedding even without knowing the true chaotic model. These recurrence patterns are where the *k*-nearest neighbors algorithm is being used as a phase space reconstruction parameter search algorithm, which is more resilient to noise and different types of chaotic dynamics (including color chaos) with stochastic components. This role is now described in detail in the first appendix.

In the second appendix, we present the equations for the major ARCH models that we tested using Python’s ARCH library. In the case of the fractionally integrated generalized ARCH model, the *d* parameter is automatically estimated by the algorithm used.

*3. The study reports high explanatory power for the full model but does not compare its performance to benchmark forecasting methods. Including a comparative analysis with traditional epidemiological or statistical models would better contextualize the added value of the topological machine learning approach and strengthen claims of methodological superiority.*

**Response:** The method applied in this article is a 3-stage method. A significant change was applied in the last stage as a consequence of the reviewer’s comment to integrate forecasting into the method.

Indeed, in the original article, available as a preprint, we were not proposing a new method for forecasting; we were applying a recent pre-existing 3-stage method for decomposing a noisy complex attractor down to IID noise, where topological machine learning is used as a search algorithm for finding embedding parameters where there is strongest topological exploitable information linking the last phase space point to the next value of the target series.

That is, we applied a rolling window topological machine learning algorithm to find for which embedding parameters there is the highest topological structure linking a reconstructed phase space dynamic to a target time series.

This method was originally proposed and applied to air traffic data and the impact of COVID-19 on air traffic and presented at an international artificial intelligence conference; after that, it was also applied to sunspot data. A full review of this method and its previous applications has now been introduced in the revised article for better clarity.

The original method has the following three stages:

- Stage 1 (reconstruction using topological machine learning): Given a time series, use the topological machine learning *k*-nearest neighbors algorithm as a search algorithm for finding an embedding that captures the topological structure of an underlying attractor, linking it to the next value of the target series for phase space reconstruction. The use of topological machine learning with a rolling window is necessary at this first stage due to the type of task that is being performed, which is to find the best way to capture the local topological structure of the attractor in a way that links the attractor’s dynamics to the target series’ next value. So prediction here is about finding an exploitable topological structure in the dynamics; the goal is to find which embedding leads to the strongest topological information linking a phase point to the next value of the series.
- Stage 2 (phase space dynamics characterization): To study the reconstructed attractor and characterize its dynamics, which involves the study of markers of chaos in the original reconstructed attractor and in the filtered reconstructed attractor using the topological adaptive agent’s predictions as an adaptive noise filter.
- Stage 3 (decomposition down to IID noise): Extracting out the predictions to focus on the residuals (the noise process) and study those residuals fitting a stochastic model down to IID noise to capture the full pattern.

The reported final *R*^2^ that we presented for the preprint version was a goodness-of-fit metric rather than an out-of-sample forecasting score because the residuals’ stochastic process was researched and estimated using the full sample.

Now, while this is what we applied in the original preprint article, we made a few changes in the revised article, reflecting the reviewer’s comment, which opened up the possibility of adapting the method for epidemiological forecasting, thus adding value for epidemiological surveillance and risk analysis.

This is a different approach, implying the need for a change at the decomposition stage (third stage of the method). In this instance, rather than using the filtered residuals with the *k*-nearest neighbors predictions used as an adaptive topological forward-looking noise filter and fitting a time series model to the resulting noise down to IID components, we changed the third stage of the model to test different forecasting schemes that can be implemented using the reconstructed attractor and the resulting residuals to capture the full process down to IID noise in a way that allows for forecasting and supports risk analysis and epidemiological surveillance rather than just epidemiological characterization, which was the original intention of the article.

In this way, while the first two stages of the method are implemented as in the original method, the third stage has been changed to incorporate a forecasting-based decomposition that provides value for epidemiological surveillance since the decomposition stage directly targets surveillance and risk analysis while at the same time covering the modeling component.

To perform a decomposition that can be turned into an epidemiological surveillance scheme and to investigate the degree to which the reconstructed attractor can support epidemiological surveillance without losing the decomposition and modeling dimension, that is, the point of the last stage was the critical change that led to a more extensive revision of the empirical section at the last stage of the method.

The revised article in the Results section now features four subsections: the first is the characterization of the surveillance series with the signal spectral analysis, and subsections three to four correspond to the three stages of the method.

It is at the last stage that we implemented the switch from the original method, which was developed for “dynamics characterization,” to a “dynamics characterization and forecasting” approach.

In this case, we test different prediction algorithms on the reconstructed attractor using the same rolling window as the one used for the adaptive *k*-nearest neighbors model and use the *k*-nearest neighbors as a benchmark to get a baseline on topological predictability on multiple forecasting horizons.

The main point at the first part of the third stage, before decomposition, is now to find the degree to which the attractor supports prediction on different horizons and which models work best for which horizons, covering different major families of models.

These models are, thus, first tested on prediction on a 1 week ahead prediction horizon using the reconstructed attractor, and then they are also tested on multiple weeks ahead prediction to evaluate how the attractor can support prediction on different horizons and which algorithms should be used.

It becomes evident from the results that there is no single best algorithm that works for all horizons. This is due to the nature of the attractor; thus while for short horizons we get best results from one algorithm, for longer horizons, we have to switch to another algorithm for forecasting and epidemiological surveillance.

After addressing this part of the forecasting issue, which is still within the context of characterizing the nature of the chaotic dynamics but at the same time has direct implications for epidemiological surveillance, we proceed to the decomposition process using the best performer in the one period ahead forecasting and using the residuals to address the stochastic component.

In this last stage, again, we introduce a change to the original method and article to best fit the forecasting problem to get a better fit into epidemiological surveillance rather than just epidemiological modeling. Therefore, we no longer estimate a model on the full sample residuals, which is what is done in the original method that is aimed at supporting stochastic model testing on a filtered noise term rather than forecasting.

Instead, we now evaluate a dynamical method that does not require the full sample and that can be integrated into the adaptive framework of the article and of the resulting epidemiological surveillance process.

Specifically, we still find nonstationary variance with ARCH signatures in the residuals of the best performer in the one-period forecasting, so we implement and test an EWMA ARCH model, which is better suited for the forecasting system that is used in the rolling window machine learning framework. Additionally, we test whether the EWMA algorithm is capable of leading to IID noise and of fully capturing the nonstationary variance component consistent with the forecasting scheme, which it does.

We also compare the EWMA’s performance with major ARCH models, showing that neither of these models leads to IID noise. With this modification, the method used not only allows us to decompose the attractor down to IID noise (original purpose of the method) but also allows us to draw direct insights into epidemiological surveillance and risk analysis in a more effective way than in the original preprint version.

The last stage of the model is thus renamed from decomposition to predictive decomposition to highlight the change to the original method. The references to the original three-stage method are presented, and this change to the method is now addressed in the revised article.

It is important to stress that the comparison of the prediction algorithms tested in the third stage of the method cannot be moved to the first stage of the method with the algorithms used for deciding the phase space embedding. Since the first stage of the method is not about a comparison of prediction algorithms, instead it is about finding which embedding captures the strongest topological structure linking the phase space trajectory to the time series, so a topological machine learning unit has to be used in the first stage of the method. The employment of *k*-nearest neighbors for this is effective since it is a noise-resilient algorithm, making it more appropriate when dealing with stochastic chaos. This explanation is now integrated for greater clarity into the Methods section as well as in Appendix A.

In the last stage of the method, different algorithms can indeed be tested, since this is a decomposition stage rather than an embedding selection stage. So the inclusion of these different algorithms makes the predictive decomposition and, in this way, adds value from an epidemiological surveillance standpoint, which now constitutes the third stage of the modified method.

## Round 2 Review

### Reviewer F

#### General Comments

*This paper presents an interesting and technically rich application of chaos theory, nonlinear time series analysis, and topological machine learning to influenza epidemiological data. The methodology is strong, and the topic is relevant for epidemiological modeling and risk analysis.*

*The authors have made some efforts to improve the manuscript following the first round of review, particularly in terms of organization and presentation. However, while progress has been made, several of the initial comments remain only partially addressed, and further revisions are required.*

#### Specific Comments

##### Major Comments

*1. Although some improvements in clarity are noticeable, the manuscript remains dense and difficult to follow, particularly in the Abstract, Methods, and Results sections.*

**Response:** The authors thank the reviewer for the suggestions for streamlining the content for the Methods and Results sections.

The paper now submitted has been significantly reduced in length in the Methods and Results section, with the changes marked in red.

*2. The Abstract remains too long and should be further simplified and condensed.*

**Response:** The abstract has been revised in a few sections to cut redundancies, keeping, however, the editor’s requirement for a structured abstract.

*3. The structure of the Methods section could still be improved. The addition of a clear workflow diagram summarizing the methodology would greatly enhance readability.*

**Response:** The Methods section was restructured, now opening with a workflow figure and a subsection for each of the method’s three steps, describing each step in terms of the reason for the step and the main work done in it, the objective, the hypothesis, the statistical techniques used, and the output.

*4. The Results section continues to lack sufficient interpretation of figures and tables. The authors should more clearly explain what each result shows and why it is important.*

**Response:** The Results section has been reduced to include the direct interpretation of the results in the context of the techniques being used, focusing more directly on these statistical techniques and the results presented in each table and figure.

## Abbreviations

ARCH: autoregressive conditional heteroskedasticity
EWMA: exponentially weighted moving average
IID: independent and identically distributed
